# Prevalence and risk factors of patients with chronic bronchitis among Iraqi adults

**DOI:** 10.25122/jml-2022-0284

**Published:** 2023-03

**Authors:** Ali Hussein Abbas, Mohammed Abdulkareem Mustafa, Mohammed Abozaid

**Affiliations:** 1 Community Health Nursing Department, College of Nursing, University of Al-Muthanna, Al-Muthanna, Iraq; 2 Adult Nursing Department, College of Nursing, University of Al-Kufa, Kufa, Iraq; 3 Department of Chest Disease, Faculty of Medicine, Zagazig University, Zagazig, Egypt

**Keywords:** risk factors, chronic bronchitis, demographic characteristics

## Abstract

This study aimed to identify the risk factors associated with chronic bronchitis among patients seeking medical attention for respiratory conditions in Al-Najaf Al-Ashraf city, Iraq. The study employed a case-control design and recruited 134 participants using convenient sampling. Data was collected using a questionnaire consisting of four parts which included demographic characteristics, individual factors, family history, and seasonal, environmental, and nutritional factors. The majority of participants were males aged between 21 and 35 years, with 71.8% of the study group residing in rural areas and 66.3% of the control group living in urban areas. We found that asthma was the most prevalent associated disease among chronic bronchitis patients, with 64.1% reporting it. The risk factors associated with chronic bronchitis were residency, smoking, exposure to secondhand smoke, respiratory sensitivity, dust sensitivity, spring sensitivity, hay fever, asthma, pulmonary obstruction, pneumonia, pertussis, and family history. The study highlights the need for smoking cessation, physical fitness, and healthy eating habits to prevent chronic bronchitis. The findings of this study are important for healthcare professionals in Iraq to design and implement effective prevention and management strategies for chronic bronchitis.

## INTRODUCTION

Chronic bronchitis (CB) is a form of chronic obstructive pulmonary disease (COPD) characterized by a productive cough lasting more than three months and recurring over two years. Patients with CB often experience symptoms such as chest or stomach pain, lethargy, and a chronic productive cough and are frequently hospitalized [1]. Individuals with chronic bronchitis or chronic obstructive pulmonary disease (COPD) may develop recurring exacerbations accompanied by increased sputum volume, purulence, or both. Reducing exacerbations is essential to decrease personal and medical expenses. Mucolytics are oral medications that decrease sputum viscosity, promoting expectoration and reducing the frequencies of COPD exacerbations [2]. Bronchitis is associated with chronic inflammation in the airways, leading to acute respiratory events, such as exacerbations, airflow restriction, progressive loss of lung function, and, eventually, a higher all-cause mortality rate [3]. The prevalence of chronic bronchitis in the general adult population ranges from 3.6% to 22% globally and 5.5% to 7.2% in Sweden.

Additionally, smokers with COPD have an even higher prevalence, ranging from 19% to 74%. Studies on chronic bronchitis have primarily focused on elderly individuals and those with COPD, leading to a lack of research on young individuals despite their increased incidence of the disease. Most of these studies involve individuals between 18 and 40 years old, and the prevalence rates are typically between 1% and 10% [4].

## MATERIAL AND METHODS

A case-control design was employed to achieve the objective of this study. The study was conducted from January 1st to May 25th, 2022, after obtaining official authorization from the Al-Najaf Al-Ashraf Health Directorate. The study population included 134 patients who sought medical care for respiratory problems at Al-Najaf Al-Ashraf hospitals and served as a convenient sample. The study questionnaire was then distributed to each participant, and they were requested to complete the questionnaire, which typically took 10 to 20 minutes. The questionnaire was developed by the researchers based on previous studies, textbooks, personal experience, and expert opinions and included four sections. Part 1 contains seven components related to demographic characteristics, including gender, age, level of education, residence, occupation, smoking, and exposure to smoking. Part 2 consists of eight items related to individual factors, such as height, weight, body mass index, and sensitivity to allergies, including hay fever, respiratory sensitivity, dust sensitivity, and spring sensitivity. Part 3 contains two categories: family history and five chronic diseases (asthma, lung obstruction, pneumonia, and pertussis). Part 4 includes eight elements related to seasonal, environmental, and nutritional factors, such as summer, winter, mass burning, dust, air pollutants, seasonal allergies, spicy foods, and egg consumption). Finally, participants were requested to complete the questionnaire themselves, and researchers gathered height and weight measurements using appropriate scales. The data-gathering process for each participant ranged from 10 to 20 minutes.

## RESULTS

Table 1 shows the distribution of study participants according to demographic data. The mean age of participants was 34.48 years, which was similar between the study group (48%) and the control group (47%). Gender was equally distributed in both groups, with half of the participants being male. Educational level varied between the groups, with 33.3% of the study group having a middle school education and 36.8% of the control group having a college education. Place of residence was also different between the groups, with 71.8% of the study group living in rural areas and 66.3% of the control group in urban areas. Most participants in both groups were employed, with 35.9% in the study group and 37.9% in the control group. Most participants in both groups were exposed to secondhand smoke, with 84.6% in the study group and 66.3% in the control group reporting exposure. The study revealed that half of the study sample and 85.3% of the control group were nonsmokers.

**Table 1:** Demographic characteristics of study and control groups.

Variables	Rating	Statistic	Group	
			Study	Control
Age group	≤20	Freq.	6	17
		%	15.4%	17.9%
	21-35	Freq.	19	45
		%	48.7%	47.4%
	36-50	Freq.	7	21
		%	17.9%	22.1%
	51-65	Freq.	2	6
		%	5.1%	6.3%
	66-80	Freq.	4	6
		%	10.3%	6.3%
	81+	Freq.	1	0
		%	2.6%	0.0%
Mean±SD			34.48±1.5	
Gender	Male	Freq.	23	48
		%	59.0%	50.5%
	Female	Freq.	16	47
		%	41.0%	49.5%
Educational level	Illiterate	Freq.	6	13
		%	15.4%	13.7%
	Read and write	Freq.	2	3
		%	5.1%	3.2%
	Primary school	Freq.	10	12
		%	25.6%	12.6%
	Middle school	Freq.	13	26
		%	33.3%	27.4%
	Institute	Freq.	3	6
		%	7.7%	6.3%
	College	Freq.	5	35
		%	12.8%	36.8%
Place of residence	Rural	Freq.	28	32
		%	71.8%	33.7%
	Urban	Freq.	11	63
		%	28.2%	66.3%
Occupation	Employed	Freq.	14	36
		%	35.9%	37.9%
	Freelancer	Freq.	10	23
		%	25.6%	24.2%
	Retired	Freq.	2	0
		%	5.1%	0.0%
	Student	Freq.	6	20
		%	15.4%	21.1%
	Housewife	Freq.	7	16
		%	17.9%	16.8%
Exposure to smoking	Yes	Freq.	33	32
		%	84.6%	33.7%
	No	Freq.	6	63
		%	15.4%	66.3%
Smoking	Yes	Freq.	19	14
		%	48.7%	14.7%
	No	Freq.	20	81
		%	51.3%	85.3%

Freq. – frequency; % – Percentage; S.D – Standard deviation.

Table 2 presents the individual factors and health characteristics of participants in the study and control groups. The control group had a higher percentage of participants with normal BMI (43.2%) than the study group, where 35.9% were overweight. The study group had a significantly higher proportion of participants reporting respiratory sensitivity (84.6%) than the control group (29.5%). A similar pattern was observed with dust sensitivity, where the study group had a much higher percentage of participants reporting sensitivity to dust (87.2%) compared to the control group (41.1%). The study group also had a higher percentage of participants reporting sensitivity to spring allergens (64.1%) compared to the control group (22.1%). Finally, hay fever was reported by 66.7% of the study group, whereas only 8.4% of the control group reported experiencing this condition.

**Table 2:** Individual factors and health characteristics of study and control groups.

Variables	Rating	Statistics	Group	
			Study	Control
BMI	Underweight	Freq.	5	3
		%	12.8%	3.2%
	Normal weight	Freq.	12	41
		%	30.8%	43.2%
	Overweight	Freq.	14	34
		%	35.9%	35.8%
	Obese	Freq.	4	17
		%	10.3%	17.9%
	Morbid obesity	Freq.	4	0
		%	10.3%	0.0%
Mean±SD			26.42±6.12	
Respiratory sensitivity	Yes	Freq.	33	28
		%	84.6%	29.5%
	No	Freq.	6	67
		%	15.4%	70.5%
Dust sensitivity	Yes	Freq.	34	39
		%	87.2%	41.1%
	No	Freq.	5	56
		%	12.8%	58.9%
Spring sensitivity	Yes	Freq.	25	21
		%	64.1%	22.1%
	No	Freq.	14	74
		%	35.9%	77.9%
Hay fever	Yes	Freq.	26	8
		%	66.7%	8.4%
	No	Freq.	13	87
		%	33.3%	91.6%

Freq. – frequency; % – Percentage; S.D – Standard deviation.

Table 3 shows the distribution of study participants with chronic diseases, with a higher percentage in the study group compared to the control group. The study group had a higher percentage of participants with asthma (64.1% vs. 26.3%) and a lower percentage without pulmonary obstruction (69.2% vs. 97.9%). Regarding pneumonia, 51.3% in the study group and 91.6% in the control group did not have pneumonia. Most participants in both groups did not have pertussis, with only 15.4% in the study group and 1.1% in the control group reporting this condition. Finally, 76.9% of the study group had a family history of chronic diseases, compared to 35.8% in the control group.

**Table 3:** Respiratory chronic diseases and family history of study and control groups.

Variables	Rating	Statistics	Group	
			Study	Control
Asthma	Yes	Freq.	25	25
		%	64.1%	26.3%
	No	Freq.	14	70
		%	35.9%	73.7%
Pulmonary obstruction	Yes	Freq.	12	2
		%	30.8%	2.1%
	No	Freq.	27	93
		%	69.2%	97.9%
Pneumonia	Yes	Freq.	19	8
		%	48.7%	8.4%
	No	Freq.	20	87
		%	51.3%	91.6%
Pertussis	Yes	Freq.	6	1
		%	15.4%	1.1%
	No	Freq.	33	94
		%	84.6%	98.9%
Family History	Yes	Freq.	30	34
		%	76.9%	35.8%
	No	Freq.	9	61
		%	23.1%	64.2%

Freq. – frequency; % – Percentage.

Table 4 highlights the prevalence of allergies in both study groups. Seasonal allergy was reported by 48.7% of the study group, in contrast to only 4.2% of the control group. Environmental allergy, including multiple types of allergies, was more prevalent in the study group, with 43.6% reporting four types of allergies compared to only 2.1% in the control group. Similarly, dietary allergies were more common in the study group, with 61.5% reporting two types of allergies compared to 50.5% in the control group.

**Table 4:** Seasonal, environmental, and dietary allergies in study and control groups.

Variables	Rating	Statistics	Grouping	
			Study	Control
Seasonal allergy	No	Freq.	0	36
		%	0.00%	37.90%
	Summer	Freq.	6	12
		%	15.40%	12.60%
	Winter	Freq.	14	43
		%	35.90%	45.30%
	Both	Freq.	19	4
		%	48.70%	4.20%
Environmental	No	Freq.	1	25
		%	2.60%	26.30%
	One type of allergy	Freq.	6	41
		%	15.40%	43.20%
Allergy	Two types of allergies	Freq.	4	17
		%	10.30%	17.90%
	Three types of allergies	Freq.	11	10
		%	28.20%	10.50%
	Four types of allergies	Freq.	17	2
		%	43.60%	2.10%
Dietary allergy	No	Freq.	5	41
		%	12.80%	43.20%
	One type of allergy	Freq.	24	48
		%	61.50%	50.50%
	Two types of allergies	Freq.	10	6
		%	25.60%	6.30%

Freq. – frequency; % – Percentage.

Table 5 indicates no significant differences in age, gender, and education level among participants with chronic bronchitis. However, living in a rural area was associated with a significantly higher risk of chronic bronchitis, with a 3.14 times greater risk compared to those living in urban areas (p-value 0.05). Smokers were at a significantly higher risk of chronic bronchitis, with a 2.91 times greater risk compared to nonsmokers (p-value 0.05). Furthermore, exposure to secondhand smoke was associated with a significantly higher risk of chronic bronchitis, with a 5.84 times greater risk compared to non-exposure (p-value 0.05).

**Table 5:** Risk factors associated with chronic bronchitis.

Risk factors	Rating	Group		OR (CI 95%)	Chi-square (χ^2^) P-value
		Study	Control		
Age	30 and less	21	52	0.97	χ^2^=009^a^ P. 0.9 F. 1 NS
	31 and more	18	43		
Gender	Male	23	48	1.28	χ^2^=792^a^ P. 0.3 F. 0.4 NS
	Female	16	47		
Educational level	No education	6	13	1.10	χ^2^=066^a^ P. 0.7 F. 0.4 NS
	Education	33	82		
Place of residence	Rural	28	32	3.14	χ^2^=16.241^a^ P.0.000 F. 0.000 HS
	Urban	11	63		
Smoking	Yes	19	14	2.91	χ^2^=17.200^a^ P. 0.000 F. 0.000 HS
	No	20	81		
Exposure to secondhand smoke	Yes	33	32	5.84	χ^2^=28.714 P. 0.000 F. 0.000 HS
	No	6	63		

OR. in (CI 95%) – Odd Ratio at Confidence Interval (95%); χ^2^ – Chi-Square Value, P. – P-value of Pearson Chi-Square; F. – P-value for Fisher exact; HS. – Highly Significant; NS. – Non-Significant.

There was no association between body mass index and chronic bronchitis (Table 6). However, patients with respiratory sensitivity had a 6.58 times higher risk of developing chronic bronchitis (p= =0.05). Additionally, participants with dust sensitivity had a 5.68 times higher risk of chronic bronchitis (p=0.05), while those with spring sensitivity had about a 3.42 times higher risk of chronic bronchitis (p=0.05). Finally, patients with hay fever had a 5.88 times higher risk of developing bronchitis at (p=0.05).

**Table 6:** Individual and environmental risk factors associated with chronic bronchitis.

Possible risk factors	Rating	Group		OR (CI 95%)	Chi-square (χ^2^) P-value
		Study	Control		
BMI	Abnormal weight	27	54	1.47	χ^2^=1.775^a^ P. 0.183 F. 0.243 NS
	Normal weight	12	41		
Respiratory sensitivity	Yes	33	28	6.58	χ^2^=33.900^a^ P. 0.000 F. 0.000 HS
	No	6	67		
Dust sensitivity	Yes	34	39	5.68	χ^2^=23.722^a^ P. 0.000 F. 0.000 HS
	No	5	56		
Spring sensitivity and breath	Yes	25	21	3.42	χ^2^=21.632^a^ P. 0.000 F. 0.000 HS
	No	14	74		
Hay fever	Yes	26	8	5.88	χ^2^=49.538^a^ P. 0.000 F. 0.000 HS
	No	13	87		

OR. in (CI 95%) – Odd Ratio at Confidence Interval (95%); χ^2^ – Chi-Square Value, P. – P-value of Pearson Chi-Square; F. – P-value for Fisher exact; HS. – Highly Significant; NS. – Non-Significant.

There was a high correlation between clinical information and chronic bronchitis (Table 7). Participants with asthma had a roughly 3.00 times higher risk of developing chronic bronchitis (p-value=0.05). Those with pulmonary obstruction had approximately 3.81 times the risk of chronic bronchitis (p-value=0.05). Participants with pneumonia had about 3.76 times the risk of chronic bronchitis (p-value=0.05), while those with pertussis had a 3.30 times higher risk (p-value=0.05). Family history was also a significant risk factor, with patients having approximately 3.65 times the risk of chronic bronchitis (p-value=0.05).

**Table 7:** Clinical risk factors associated with chronic bronchitis.

Risk factors	Rating	Group		OR (CI 95%)	Chi-square (χ^2^), P-value
		Study	Control		
Asthma	Yes	25	25	3.00	χ^2^=16.878^a^ P. 0.000 F. 0.000 HS
	No	14	70		
Pulmonary obstruction	Yes	12	2	3.81	χ^2^=24.280^a^ P. 0.000 F. 0.000 HS
	No	27	93		
Pneumonia	Yes	19	8	3.76	χ^2^=27.905^a^ P. 0.000 F. 0.000 HS
	No	20	87		
Pertussis	Yes	6	1	3.30	χ^2^=11.471^a^ P. 0.001 F. 0.002 HS
	No	33	94		
Family history	Yes	30	34	3.65	χ^2^=18.750^a^ P. 0.000 F. 0.000 HS
	No	9	61		

OR. in (CI 95%) – Odd Ratio at Confidence Interval (95%); χ^2^ – Chi-Square Value, P. – P-value of Pearson Chi-Square; F. – P-value for Fisher exact; HS. – Highly Significant; NS. – Non-Significant.

Finally, there was a highly significant relationship between allergy types and chronic bronchitis in all items except dietary allergy to eggs (Table 8). Participants with seasonal allergies had a 4.38 times higher risk of developing bronchitis in the summer and about 3.94 times higher risk in the winter (p-value= 0.05). Environmental allergies to mass burning, dust, and air pollution were associated with a 3.13, 5.67, and 3.76 times higher risk of chronic bronchitis, respectively, at a p-value of 0.05. For dietary allergies, spicy food was associated with a 1.25 times higher risk of chronic bronchitis (p-value=0.05.

**Table 8:** Relationship between allergy types and chronic bronchitis.

Risk factors		Rating	Group		OR (CI 95%)	Chi-square (χ^2^) P-value
			Study	Control		
Seasonal allergy	Summer	Yes	26	16	4.38	χ^2^=31.896^a^ P. 0.000 F. 000.0 HS
		No	13	79		
	Winter	Yes	32	40	3.94	χ^2^=17.747^a^ P. 0 F. 0.000 HS
		No	7	55		
Environmental allergies	Mass Burring	Yes	27	29	3.13	χ^2^=17.027^a^ P. 0 F. 0.000 HS
		No	12	66		
	Dust	Yes	33	33	5.67	χ^2^=27.521^a^ P. 0 F. 0.000 HS
		No	6	62		
	Air pollution	Yes	30	33	3.76	χ^2^=19.753^a^ P. 0.000 F. 0.000 HS
		No	9	62		
Dietary allergies	Seasonal because of pollen	Yes	27	21	4.03	χ^2^=26.709^a^ P. 0.000 F. 0.000 HS
		No	12	74		
	Spicy	Yes	32	41	3.82	χ^2^=16.865^a^ P. 0 F. 0.000 HS
		No	7	54		
	Egg	Yes	10	19	1.25	χ^2^=0.519^a^ P. 0.471 F. 0.494 NS
		No	29	76		

OR. in (CI 95%) – Odd Ratio at Confidence Interval (95%); χ^2^ – Chi-Square Value, P. – P-value of Pearson Chi-Square; F. – P-value for Fisher exact; HS. – Highly Significant; NS. – Non-Significant.

## DISCUSSION

### Part I: Characteristics of patients with chronic bronchitis

The current study found that a significant proportion of the study group (48%) were males and fell within the age range of 21-35 years. Concerning the level of education, 33.3% of the study group completed middle school. A large percentage (71.8%) lived in rural areas, and 35.9% were employed. In addition, 84.6% lived with smokers, although half of the participants were nonsmokers. These findings are consistent with previous studies that reported similar characteristics in patients with chronic bronchitis [5-7]. For example, Abo-Elkhair et al. reported that most of their study sample were males above 20 years of age, similar to the current study [5]. Another study by Rumselly et al. aimed to investigate the factors related to bronchitis in employees of the cement warehouse unit in Ambon and reported that most of the subjects were employed. [6]. Furthermore, Siddharthan et al. also found that most of their study sample were rural people who never smoked, which is consistent with the current study finding [7].

The study found that approximately 35.9% of the study group was overweight, 84.6% of patients had respiratory sensitivity, 87.2% had dust sensitivity, 64.1% spring sensitivity, and 66.7% had hay fever. This finding agreed with a study conducted by Mejza et al. [8], and their results revealed that most patients had respiratory and dust sensitivity. Shin et al. [9] also reported that most study participants were overweight. In addition, the current study found that 64.1% of the patients in the study group had asthma, and 76.9% had a family history of allergy, which may be due to the environmental and climatic changes that occurred in Iraq in recent years in addition to genetic factors.

### Part II: Possible risk factors of patients with chronic bronchitis

We identified several risk factors for patients with chronic bronchitis, including residency area and positive/negative smoking status. Patients with respiratory sensitivity, dust sensitivity, spring sensitivity, and hay fever had a higher risk of developing chronic bronchitis (p-value=0.05). Patients with asthma, pulmonary obstruction, pneumonia, and pertussis were also found to be at a greater risk of developing chronic bronchitis (p-value=0.05). Additionally, patients with dietary allergies (eggs), seasonal allergies (summer), and environmental allergies (mass burning) were more susceptible to chronic bronchitis (p-value=0.05).

These findings are consistent with previous research, such as Long and Lai's study on chronic cough patients, which identified environmental factors, smoking, and lifestyle choices as influential factors in chronic bronchitis [10]. Wu et al. also reported that individuals with chronic bronchitis had a worse pulmonary function, higher BMI, higher allergy score, and a higher incidence of exacerbations in the previous year [11]. Similarly, Choi et al. found that patients with chronic bronchitis had higher levels of asthma symptoms and were more likely to be current smokers compared to non-chronic bronchitis patients [12].

Lung function testing in patients with chronic bronchitis revealed decreased results compared to non-chronic bronchitis patients. Exposure to air pollution was also associated with chronic bronchitis in the study by Wang et al. [13]. Pahwa et al. revealed that body mass index (obesity), exposure to environmental cigarette smoke and musty air in the home, allergy to house dust, and increasing age were the important drivers of CB. CB is more prevalent among individuals with higher household income, older age, allergies, parental history of lung disease, exposure to stubble smoke, obesity, prenatal smoking exposure, and predominantly among females [14]. Konrad et al. mentioned that the CB prevalence was greater in females (7.2%) than in males (5.0%) and that people with CB are more likely to be older, have poorer incomes, lower levels of education, and live in rural regions [15].

Furthermore, the relationship between allergic reactions to mold and wood particles encountered in the workplace may be affected by smoking. Obesity and smoking status are closely linked to chronic bronchitis (CB), with a stronger association found for obesity. More than 40% of smokers get chronic bronchitis at some point. The risk of developing chronic obstructive pulmonary disease, death, and an accelerated reduction in lung function are all linked to chronic bronchitis [16]. Ye et al. claimed that in rural patients, aging, smoking, and gender are not independent risk factors for chronic bronchitis but exposure to smoking, family history of COPD, and decreased pulmonary function [17].

Karunanayake et al. conducted a study in Switzerland to investigate the incidence of bronchitis and its associated risk factors [18]. Their findings identified several modifiable risk factors for bronchitis, such as obesity, smoking exposure, and mold or dampness in the home. The variation in risk factors for patients with chronic bronchitis can be attributed to differences in their environment and climate. Each group of individuals may have a distinct set of risk factors that are influenced by factors such as their living and working environment, indoor air quality, health habits, and genetic predispositions.

## CONCLUSION

The study provides important insights into the characteristics and possible risk factors of chronic bronchitis in Iraq. The findings suggest that chronic bronchitis is more common in males aged 21-35 and is often associated with asthma. The study also identified several risk factors for chronic bronchitis, including residence, smoking, respiratory sensitivity, dust sensitivity, spring sensitivity, hay fever, asthma, pulmonary obstruction, pneumonia, pertussis, and family history. Based on these findings, the study recommends that smoking cessation and avoidance of secondhand smoke are important steps for improving quality of life and reducing the risk of chronic bronchitis. Additionally, maintaining physical fitness through exercise and a healthy diet can help strengthen the immune system and reduce the risk of respiratory illness. Finally, the study emphasizes the importance of seeking timely treatment for acute bronchitis to prevent it from becoming more severe.

Overall, this study highlights the need for greater awareness and prevention of chronic bronchitis in Iraq and provides valuable insights that can inform public health interventions and policies to reduce the burden of this disease.
